# Selective Modulation of K^+^ Channel Kv7.4 Significantly Affects the Excitability of DRN 5-HT Neurons

**DOI:** 10.3389/fncel.2017.00405

**Published:** 2017-12-14

**Authors:** Chen Zhao, Min Su, Yingzi Wang, Xinmeng Li, Yongxue Zhang, Xiaona Du, Hailin Zhang

**Affiliations:** Department of Pharmacology, Hebei Medical University, The Key Laboratory of Neural and Vascular Biology, Ministry of Education, The Key Laboratory of New Drug Pharmacology and Toxicology, Shijiazhuang, China

**Keywords:** Kv7.4 channel, DRN, 5-HT neuron, fasudil, excitability

## Abstract

The serotonin (5-HT) system originating in the dorsal raphe nucleus (DRN) is implicated in various mood- and emotion-related disorders, such as anxiety, fear and stress. Abnormal activity of DRN 5-HT neurons is the key factor in the development of these disorders. Here, we describe a crucial role for the Kv7.4 potassium channel in modulating DRN 5-HT neuronal excitability. We demonstrate that Kv7.4 is selectively expressed in 5-HT neurons of the DRN. Using selective Kv7.4 opener fasudil and Kv7.4 knock-out mice, we demonstrate that Kv7.4 is a potent modulator of DRN 5-HT neuronal excitability. Furthermore, we demonstrate that the cellular redox signaling mechanism is involved in this 5-HT activation of Kv7.4. The current study suggests a new strategy for treating psychiatric disorders related to altered activity of DRN 5-HT neurons using K^+^ channel modulators.

## Introduction

The serotonergic (5-HT) system originating in the dorsal raphe nucleus (DRN) is involved in regulating various physiological and behavioral functions, including mood- and emotion-related behaviors (Michelsen et al., 2008; Olivier, 2015). Many observations suggest that abnormal activity of DRN neurons is linked to psychiatric disorders, such as major depression disorder (MDD) and anxiety (Robinson et al., 1989; Deakin, 1991; Graeff et al., 1996; Kinney et al., 1997; Bielau et al., 2005; Maier and Watkins, 2005). Aversive stimuli-produced stress, anxiety and fear increase neuronal activity in subpopulations of serotonergic neurons and increase serotonin levels in the vicinity of DRN neurons (Amat et al., 2005). The activity of a relatively small number of 5-HT neurons is powerfully poised to regulate the excitability of large ensembles of neural networks distributed across the entire brain (Geddes et al., 2016). Thus, activity of the 5-HT neurons needs to be well controlled to maintain emotional homeostasis. Neuronal activity is controlled by the intrinsic activity of ion channels as well as by the regulatory transmitters and hormones. For 5-HT neurons, some K^+^ channels, including SK channels (Crespi, 2010; Sargin et al., 2016), TREK1 channels (Ye et al., 2015) and Kir3/GIRK channels (Llamosas et al., 2015, 2017; Montalbano et al., 2015) have been suggested as playing important roles in controlling the intrinsic neuronal activity, and modulating these channels may alleviate emotional disorders. For example, up-regulation of SK3 channels in serotonin-producing neurons are responsible for greatly reduced activity in these neurons from a model of isolated mice; blocking these inhibitory SK3 channels restores normal activity in the serotonin-producing cells and alleviates the depressive symptoms of the isolated mice (Sargin et al., 2016). Similarly, blocking TREK1 channels substantially increases the firing rate of 5-HT-ergic neurons in the DRN and induces a significant antidepressant-like response in a rat model of depression (Ye et al., 2015). Deletion of the GIRK2 or blocking the activity of GIRK2 in DRN promotes a depression-resistant phenotype (Llamosas et al., 2015). It is clear from these and other experiments that the firing activity of 5-HT neurons in the DRN is critical to determining the role of the neurons in related behavioral functions. However, despite these advancements, complete understanding of the firing activity in the DRN and its underlying mechanisms is currently lacking. Neuronal Kv7 channels are widely expressed in the brain and the Kv7.2, Kv7.3 and Kv7.5 channels are the most abundant (Biervert et al., 1998). On the other hand, the Kv7.4 subunit has a more restricted brain regional expression, only present in discrete nuclei of the brainstem, including the DRN (Kharkovets et al., 2000; Dalby-Brown et al., 2006). Opening of Kv7 channels leads to neuronal hyperpolarization, membrane potential stabilization and decreased excitability (Hu et al., 2007; Zhang et al., 2013). This makes Kv7 channels particularly interesting as targets in various CNS diseases that are characterized by neuronal over-activity, including stress, anxiety and epilepsy (Korsgaard et al., 2005; Barrese et al., 2010). Interestingly, expression of Kv7.4 mRNA and proteins has been reported in DRN (Kharkovets et al., 2000). However, the functional role of Kv7.4 in regulating the excitability of DRN 5-HT neurons has not been elucidated. A previous study demonstrated that activation of pertussis toxin (PTX)-sensitive Gi/o-coupled receptors (Somatostatin and substance P) can augment Kv7 currents in hippocampal neurons (Moore et al., 1988) and DRG neurons (Linley et al., 2012). We hypothesize that the cellular redox signaling mechanism is involved in this 5-HT activation of Kv7.4. Consistent with this hypothesis, we found in this study that Kv7.4 is a dominant and potent modulator of DRN 5-HT neuron excitability, and a redox mechanism is involved in 5-HT-mediated activation of Kv7.4 currents in DRN 5-HT neurons.

## Materials and Methods

### Animal Preparation

The Kv7.4 knock-out mice (Kv7.4^−/−^) were kindly provided by Prof Thomas Jentsch (FMP, MDC, Berlin, Germany; Kharkovets et al., 2006). Eight to twelve week-old male C57BL/6 mice (Vital River, China) were used for the studies. Mice were housed at constant temperature and humidity, and under a 12 h:12 h light-dark cycle (with switches at 8 AM and 8 PM), with water and food available *ad libitum*. All experiments were conducted in accordance with the guidelines of Animal Care and Use Committee of Hebei Medical University and approved by the Animal Ethics Committee of Hebei Medical University.

### Electrophysiology

Brain slices containing DRN were prepared from 8 to 12-week-old male C57BL/6 mice as reported (Krishnan et al., 2007). In brief, mice were euthanized with pentobarbital sodium (200 mg/kg, i.p). Coronal brainstem slices (200 μm thickness) containing DRN (AP −3.8 to −4.8 mm; LM 0 mm; and DV −2.8 to −3.8 mm) were cut in a vibratome (VT1200S; Leica, Germany). The DRN containing slices were placed in ice-cold sucrose solution (in mM: 260 sucrose, 3 KCl, 26 NaHCO_3_, 1.25 NaH_2_PO_4_, 2 CaCl_2_, 2 MgCl_2_, 10 D-glucose, saturated with 95% O_2_/5% CO_2_) and incubated for 30 min at 37°C in oxygenated artificial cerebrospinal fluid (aCSF; in mM: 130 NaCl, 3 KCl, 26 NaHCO_3_, 1.25 NaH_2_PO_4_, 2 CaCl_2_, 2 MgCl_2_, 10 D-glucose). Then, the slices were kept for recovery at room temperature (23–25°C) for at least 1.5 h, after which they were transferred to the recording chamber and superfused with oxygenated aCSF (room temperature) at 2–3 ml/min. Glass recording pipettes (3–5 MΩ) were filled with an internal solution containing (mM): 115 K-methyl sulfate, 20 KCl, 1 MgCl_2_, 10 HEPES, 0.1 EGTA, 2 MgATP, 0.3 GTP, pH adjusted to 7.4 with KOH. DRN 5-HT neurons were identified by the presence of tryptophan hydroxylase (TPH; single-cell PCR). All recordings were performed from neurons located on the midline in ventromedial subdivisions of the DRN where 5-HT neurons were densely located as previously reported (Hioki et al., 2010). In the whole-cell recording, the recorded neurons were identified by the following functional characteristics: (i) no spontaneous firing; (ii) a large after-hyperpolarization, AHP; (iii) inhibition of the firing activity by serotonin (Aghajanian and Vandermaelen, 1982; Vandermaelen and Aghajanian, 1983). In the cell-attach recording, spontaneous activity was monitored in current-clamp mode (*I* = 0) in the presence of 10 μM phenylephrine (PE), and the recorded neurons were characterized by discharge with a slow (0.5–3 Hz), regular (clock-like) pattern and all neurons showed response to 5-HT. Kv7/M currents were recorded in the whole-cell voltage-clamp mode; the elicited firing activity was recorded in the whole-cell current-clamp mode and the spontaneous firing activity was recorded in the cell-attach mode. Data acquisition and on-line analysis were done using an EPC10 amplifier and Patchmaster software (HEKA Electronics). The Kv7/M current amplitude was measured from the peak deactivation current (tail current) elicited by a 800 ms square voltage step to −50 mV from a holding potential of −20 mV, calculated as the difference between the peak of a 20-ms segment, taken 10–30 ms into the hyperpolarizing step, and the average during the last 50 ms of that step (Zhang et al., 2016). The elicited firing was induced by a 250 pA depolarizing current for 500 ms. Access resistance (Ra) in our recordings was in the range of 5–15 MΩ. Ra was obtained directly from the reading of the EPC 10 amplifier after the whole cell capacitive currents (generated with a 10 mV hyperpolarization pulse) were compensated (Cslow compensation in EPC 10). The capacitive currents could be compensated by 90% or more, thus obtained Ra should be fairly close to the real value. The neurons in whole cell configuration with a total resistance of 20 folds larger than Ra were used for experiments; otherwise the recordings were discontinued. Also at the end of the experiments, the Cslow compensation were re-checked to make sure the compensation did not need readjustment; otherwise the neurons were discarded in further analysis. The neuron capacitance values were within the range of 20–40 pF.

### Immunohistochemistry

Mice were perfused with 20 ml of cold PBS and 20 ml of 4% paraformaldehyde (PFA). The brain was removed and post-fixed with 4% PFA at 4°C overnight; then, it was dehydrated with 30% sucrose at 4°C for 2 days. Brain tissue was sliced into coronal sections (60 μm thick) using a vibratome (Leica VT1200s, Germany). Brain sections were stored in PBS at 4°C until use. Sections were blocked with blocking buffer (3% BSA, PBS with 0.3% Triton X-100) for 1 h and 10% donkey serum for 1 h. Sections were incubated with primary rabbit anti-serotonin (Sigma 1:1000) and goat anti-Kv7.4 (Santa Cruz 1:400) diluted in 1% BSA and 0.1% Triton X-100 at 4°C overnight. The next day, sections were washed three times (10 min) in PBS and incubated with secondary antibodies, a donkey anti-rabbit TRITC affinity (1:400; Life Technologies) and donkey anti-goat FITC affinity (1:400; Life Technologies) for 2 h at room temperature. Sections were then washed three times (10 min) in PBS. DAPI stain (Sigma-Aldrich, USA) was added to wash for 15 min. Sections were washed three times before mounting and coverslipping with Prolong Gold (Life Technologies, USA). Images were acquired using a Leica TCS SP5 confocal laser microscope (Leica, Germany) equipped with laser lines for DAPI (405 Diode), FITC (Argon 488) and TRITC (DPSS 561).

### Single-Cell PCR

After electrophysiological recording, the recorded cell was harvested with gentle suction to the recording pipette and expelled into a PCR tube containing 1 μl of Oligo-dT (50 mM), 1 μl of dNTP Mixture (10 mM) and 2 μl of RNase free dH_2_O. The mixture was heated to 65°C for 5 min and then placed on ice for 1 min. Single-strand cDNA was synthesized from the cellular mRNA by adding 2 μl of 5× PrimeScript II buffer (50 mM), 0.5 μl of RNase inhibitor (40 U/μl), 1 μl of PrimeScript II RTase (200 U/μl) and 1.5 μl RNase free dH_2_O and then incubating the mixture at 50°C for 50 min. Synthesis of single-cell cDNA was performed using a C1000 Touch thermal cycler-CFX96 Real-time PCR (California, USA). First strand synthesis was executed at 95°C (5 min) followed by 30 cycles (95°C for 50 s, 56°C for 45 s and 72°C for 50 s) and a final 10 min elongation at 72°C by adding the specific “outer” primer pairs into each PCR tube (final volume 25 μl). Then, 4 μl of the product of the first PCR was used in the second amplification round by using a specific “inner” primer (final volume 50 μl). The second amplification round consisted of heating the samples to 95°C for 5 min followed by 35 cycles (95°C for 50 s, 58–62°C for 45 s and 72°C for 50 s) and 10 min elongation at 72°C. The products of the second PCR were analyzed in 2% agarose gels and stained with 10% GoldView™. PrimeScriptTM II 1st Strand cDAN Synthesis Kit and GoTaq Green Master Mix were obtained from Takara-Clontech (Kyoto, Japan) and Promega (Madison, USA), respectively.

The “outer” primers (from 5′ to 3′) were as follows:

- GAPDH (NM 008084.3):
- AAATGGTGAAGGTCGGTGTGAACG
- AGTGATGGCATGGACTGTGGTCAT
- TPH (NM 173391.3):
- GAGTCCTCATGTACGGCACC
- AGGCCGAACTCGATTGTGAA
- 5-HT_1A_ (NM 008308.4):
- CAGCCAGGTAGTGGGGACTG
- GTCTTCCTCTCACGGGCCAA
- Kv7.4 (NM 001081142.1):
- CCCGGGTGGACCAAATTGT
- AGCCCTTCAGTCCATGTTGG

The “inner” primers (from 5′ to 3′) were as follows:

- GAPDH (NM 008084.3):
- GCAAATTCAACGGCACAGTCAAGG
- TCTCGTGGTTCACACCCATCACAA
- TPH (NM 173391.3):
- TGGCTACAGGGAAGACAACG
- GTATCTGGTTCCGGGGTGTA
- 5-HT_1A_ (NM 008308.4):
- CGGTGAGACAGGGTGAGGAC
- GCGGGGACATAGGAGGTAGC
- Kv7.4 (NM 001081142.1):
- ATGGGGCGCGTAGTCAAGGT
- GGGCTGTGGTAGTCCGAGGTG

### Drugs

Serotonin was purchased from Tokyo Chemical Industry (Japan). 5-HT_1A_ receptor agonist 8-Hydroxy-DPAT hydrobromide (8-OH-DPAT) was from Abcam (Shanghai, China). 5-HT_1A_ receptor antagonist WAY-100635 and reducing agent DL-Dithiothreitol (DTT) were purchased from Sigma-Aldrich (USA). Fasudil was from the National Institute for the Control of Pharmaceutical and Biological Products (Beijing, China). XE991 dihydrochloride was from the Alomone Labs (Jerusalem, Israel). All drugs were prepared fresh on the test day.

### Statistics

All data are shown as the mean ± SEM. Statistical analysis of the same neuron was performed by the paired samples test. Statistical analysis of differences between groups was carried out by the independent samples test. For data that failed the normality test, the paired sample Wilcoxon signed rank test or Mann-Whitney tests were used. *P* values ≤ 0.05 were accepted as significant.

## Results

### Kv7.4 Channels Are Expressed in DRN 5-HT Neurons

**Figure 1:**
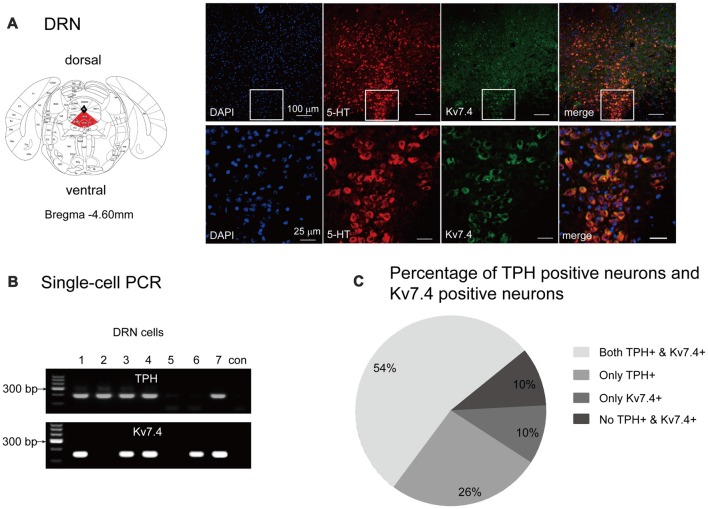
Kv7.4 channels expressed in dorsal raphe nucleus (DRN) 5-HT neurons. **(A)** Confocal images showing co-expression of DAPI (blue), Kv7.4 (green) and TPH (red) represent 5-HT neurons (up-panel, scale bar, 100 μm). Lower panel represents a zoomed-in area of DRN (scale bar, 25 μm). **(B)** Single-cell PCR from DRN cells. “con” indicates negative control in which no template was added in the RT-PCR reaction. **(C)** Percentage of 5-HT neurons (with expression of TPH) and Kv7.4 positive neurons from 61 DRN neurons.

First, we used the immunohistochemistry approach to determine the Kv7.4 protein distribution within murine DRN regions. We observed strong expression of the Kv7.4 channels in DRN 5-HT neurons (marked by anti-serotonin antibody), especially those in the ventral part (DRV; Figure 1A). We next used the single-cell PCR method to identify the proportion of Kv7.4 positive neurons in TPH positive neurons that were regarded as 5-HT neurons (Figure 1B). The results show that Kv7.4 positive neurons accounted for approximately 67% of the 5-HT neurons in the DRN (Figure 1C).

### Fasudil Potently Augments Kv7.4 Currents in DRN 5-HT Neurons

**Figure 2:**
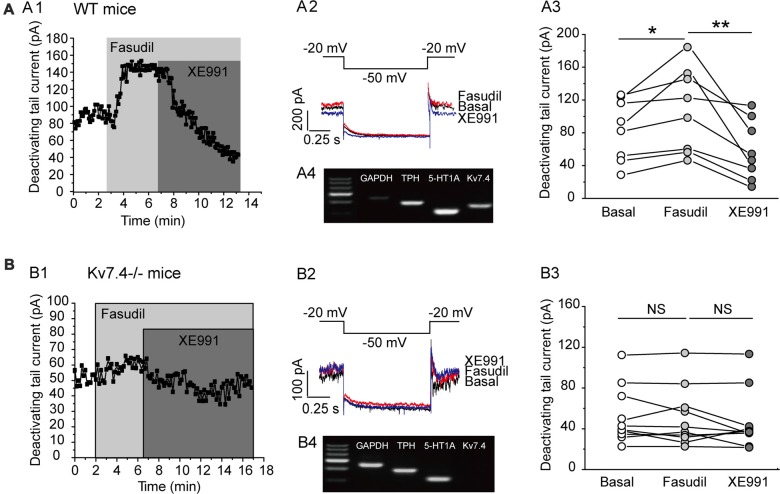
Fasudil potently augments Kv7.4 currents in DRN 5-HT neurons. **(A1)** Sample time course shows that fasudil (30 μM) augments Kv7 current, which is completely blocked by XE991 (3 μM) in neurons from WT mice. **(A2)** Voltage clamping protocol and the corresponding current traces recorded from **(A1)**. **(A3)** Summarized data for the experiment shown in **(A1)** (Wilcoxon Singed Ranks Test, *P < 0.05, *n* = 8, *N* = 5). **(A4)** Single-cell PCR result shows that the recorded neuron is a 5-HT neuron with expression of Kv7.4 and 5-HT_1A_. **(B1)** Sample time course shows that fasudil (30 μM) has no effect on Kv7 current in Kv7.4^−/−^ mice. **(B2)** Voltage clamping protocol and the corresponding current traces recorded from **(B1)**. **(B3)** Summarized data for the experiment shown in **(B1)** (*n* = 10, *N* = 5). **(B4)** Single-cell PCR result shows that the recorded neuron is a 5-HT neuron with 5-HT_1A_ expression and no Kv7.4 expression. **P < 0.01.

Previous work by our group demonstrated that fasudil is a selective Kv7.4 and Kv7.4/Kv7.5 opener and does not affect Kv7.2 and Kv7.2/Kv7.3 currents (Zhang et al., 2016). We thus evaluated the effect of fasudil on the Kv7 currents of DRN 5-HT neurons in the DRN slices from adult C57BL/6 male mice (8–12 weeks). In this experiment, the electrophysiological recordings were made on the neurons located on the midline in the ventromedial subdivision of the DRN which is most densely populated by 5-HT neurons (Paxinos and Watson, 2007; Gocho et al., 2013). The cell types (5-HT vs. non-5-HT) were identified via single-cell PCR (Figures 2A4,B4). The Kv7/M currents were recorded using the protocol shown in Figures 2A2,B2; the Kv7/M currents were measured as the characteristic slow deactivating tail currents at −50 mV step from a depolarized potential of −20 mV (Zhang et al., 2016). Bath-application of fasudil (30 μM) significantly augmented the Kv7.4 currents (from 90.7 ± 40.0 pA to 109.9 ± 51.0 pA, *n* = 8, *P = 0.012, Wilcoxon Singed Ranks Test) in DRN 5-HT neurons and the augmentation was completely reversed by XE991 (3 μM; Figures 2A1,A3). Deletion of Kv7.4 (Kv7.4^−/−^ mice) had a trend to reduce the basal Kv7/M currents (from 90.69 ± 40.03 pA to 54.9 ± 28.01 pA, *n* = 8–10, Mann-Whitney *U* = 62, *P* = 0.056) and completely abolished the augmentation of fasudil on Kv7 currents in DRN 5-HT neurons (Figures 2B1,B3). These results strongly suggest that: (i) fasudil selectively activates Kv7.4; and (ii) Kv7.4 is the functionally dominant Kv7 channel subunit in DRN 5-HT neurons. In these experiments the effect of fasudil was measured at a “standard” 4 min after application while the effect of XE 991 was measured 5 min after application in neurons from wild and Kv7.4 knockout mice.

### Kv7.4 Activity Modulates the Excitability of the DRN 5-HT Neurons

**Figure 3:**
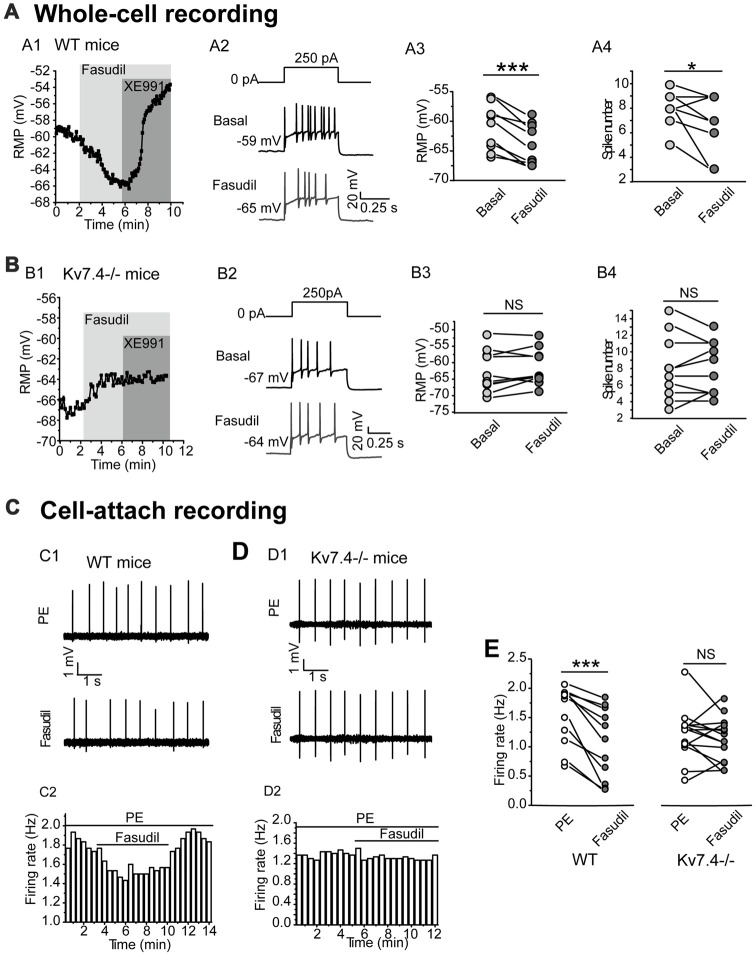
Kv7.4 activity modulates the excitability of the DRN 5-HT neurons. **(A,B)** Effects of fasudil on the elicited firing activity of DRN 5-HT neurons from WT mice and Kv7.4^−/−^ mice. **(A1)** Fasudil (30 μM) significantly hyperpolarized resting membrane potential (RMP) and the effect completely reversed by XE991 (3 μM) in WT mice. **(A2)** Typical traces with firing activity elicited by depolarizing 250 pA current injection, recorded from a neuron of WT mice. **(A3)** Summary of RMP in response to fasudil (30 μM) in WT DRN 5-HT neurons (paired samples test, ***P < 0.001, *n* = 9, *N* = 6). **(A4)** Summary of the spike number in the response of fasudil (30 μM) in WT DRN 5-HT neurons (Wilcoxon Signed Ranks Test, *P < 0.05, *n* = 9, *N* = 6). **(B1)** Fasudil (30 μM) had no significant effect on RMP of neurons from Kv7.4^−/−^ mice. **(B2)** Typical trace with firing activity elicited by depolarizing 250 pA current injection, recorded from a neuron of Kv7.4^−/−^ mice. **(B3)** Summary of RMP in response to fasudil (30 μM) in Kv7.4^−/−^ DRN 5-HT neurons (*n* = 10, *N* = 6). **(B4)** Summary of the spike number in response to fasudil (30 μM) in Kv7.4^−/−^ DRN 5-HT neurons (*n* = 10, *N* = 6). **(C)** Cell-attach recording of spontaneous firing in response to fasudil from DRN neurons of WT and Kv7.4^−/−^ mice. **(C1,C2)** Fasudil (30 μM) reduced the spontaneous firing frequency in WT DRN 5-HT neurons. **(D1,D2)** Fasudil (30 μM) had no significant effect on the spontaneous firing frequency in Kv7.4^−/−^ DRN 5-HT neurons. **(E)** Summary of effect of fasudil on phenylephrine (PE)-induced spontaneous firing rate in neurons from WT and Kv7.4^−/−^ mice (Wilcoxon Signed Ranks Test, ***P < 0.001, *n* = 11–14, *N* = 5).

We next tested the effect of fasudil on the excitability of the DRN 5-HT neurons. For this, the resting membrane potential (RMP) and the elicited spike firing of 5-HT neurons in response to a depolarizing current (250 pA) injection were recorded under the whole-cell current clamp. We found that fasudil (30 μM) strongly reduced the excitability of DRN 5-HT neurons; fasudil (30 μM) significantly hyperpolarized RMP (from −61.4 ± 3.8 mV to −64.0 ± 3.4 mV, *n* = 9, ***P = 1.88E-4, paired samples test) in neurons from WT mice (Figures 3A1–A3) and had no significant effect on RMP of neurons from Kv7.4^−/−^ mice (Figures 3B1–B3). Fasudil (30 μM) also reduced neuronal discharge induced by the depolarizing current. In WT neurons, the mean spike number during 500 ms was 8.0 ± 1.4, while after fasudil application, it was 6.7 ± 2.3 (*n* = 9, *P = 0.031, Wilcoxon Signed Ranks Test; Figures 3A2,A4). Fasudil did not affect neuronal discharge in Kv7.4^−/−^ mice (Figures 3B2,B4).

Then, we used a non-invasive loose-seal cell-attached method to record the spontaneous firing of DRN 5-HT neurons (Mlinar et al., 2016). It is known that serotonergic neurons are not intrinsic pacemakers and that they depend on extrinsic inputs to drive their firing. The noradrenergic inputs acting on α1-adrenergic receptors are thought to be a key player in this process (Gallager and Aghajanian, 1976; Baraban and Aghajanian, 1980; Menkes et al., 1981). Therefore, in the following experiments, an agonist of α1 adrenergic receptor phenylephrine (PE, 10 μM) was used to induce the spontaneous firing of 5-HT neurons in DRN brain slices. Using this method, the contribution of Kv7.4 modulation to the excitability of DRN5-HT neurons was further investigated. Fasudil significantly reduced the average firing frequency of DRN 5-HT neurons from 1.52 ± 0.5 Hz to 1.06 ± 0.6 Hz (*n* = 11, ***P = 9.77E-4, Wilcoxon Signed Ranks Test) in neurons from WT mice (Figures 3C1,C2,E) and had no effect on the firing frequency in neurons from Kv7.4^−/−^ mice (Figures 3D1,D2,E). These results suggest that Kv7.4 activity is a potent modulator in the excitability of DRN 5-HT neurons.

### Role of Kv7.4 in Serotonin-Mediated Modulation of Neuron Excitability

**Figure 4:**
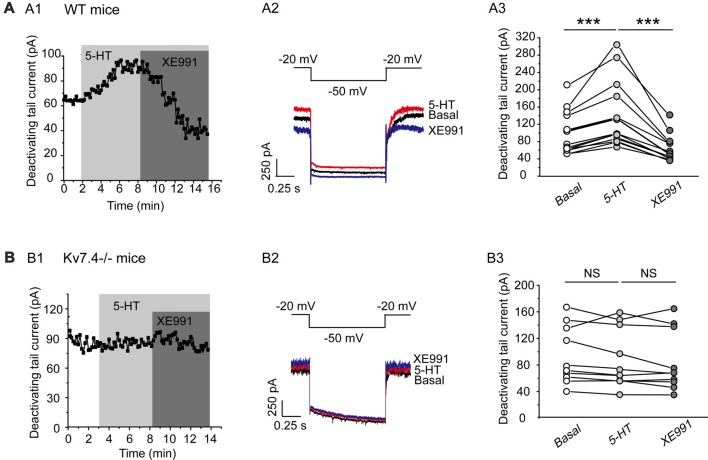
5-HT augments Kv7.4 currents in DRN 5-HT neurons. **(A1)** Sample time course shows that 5-HT (30 μM) induced a sharp augmentation of Kv7 current and the augmentation was completely reversed by XE991 (3 μM) in DRN 5-HT neurons of WT mice. **(A2)** Voltage clamping protocol and representative current traces recorded from **(A1)**. **(A3)** Summarized data for experiments shown in **(A1)** (Wilcoxon Signed Ranks Test, ***P < 0.001, *n* = 14, *N* = 6). **(B1)** Sample time course shows that 5-HT did not affect Kv7 current in neurons from Kv7.4^−/−^ mice. **(B2)** Voltage clamping protocol and representative current traces recorded from **(B1)**. **(B3)** Summarized data for experiments shown in **(B1)** (*n* = 10, *N* = 6).

In this part, we tested our hypothesis that Kv7.4 (like GIRK) could also be a regulatory target of the activated 5-HT_1A_ signaling pathway. Bath-application of 5-HT (30 μM) induced a sharp augmentation of Kv7 currents, which were completely reversed by XE991 (3 μM) in DRN 5-HT neurons of WT mice (*n* = 14, ***P = 0.001, Wilcoxon Signed Ranks Test; Figure 4A). 5-HT did not affect the Kv7 currents in Kv7.4^−/−^ mice (Figure 4B). Again as mentioned above we measured the effects of 5-HT and other drugs at a standard time (5 min for all drugs after application) in both wild and Kv7.4 knockout mice in this and also the following series of experiments.

**Figure 5:**
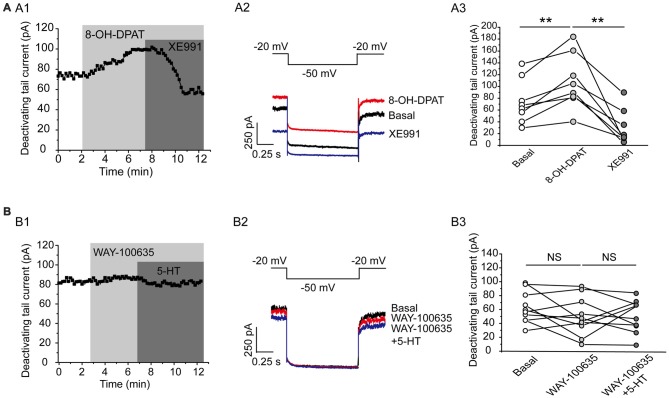
Kv7.4 is a regulation target of 5-HT_1A_ auto-inhibition. **(A1)** 5-HT_1A_ receptor agonist 8-OH-DPAT increased the Kv7 current in DRN 5-HT neurons of WT mice and the effect was reversed by XE991. **(A2)** Voltage clamping protocol and the corresponding traces recorded from **(A1)**. **(A3)** Summarized data for experiments shown in **(A1)** (paired samples test, **P < 0.01, *n* = 8, *N* = 5). **(B1)** 5-HT_1A_ receptor antagonist WAY-100635 abolished the 5-HT-induced augmentation of Kv7 current. **(B2)** Voltage clamping protocol and representative current traces recorded from **(B1)**. **(B3)** Summarized data for experiments shown in **(B1)** (*n* = 10, *N* = 6).

**Figure 6:**
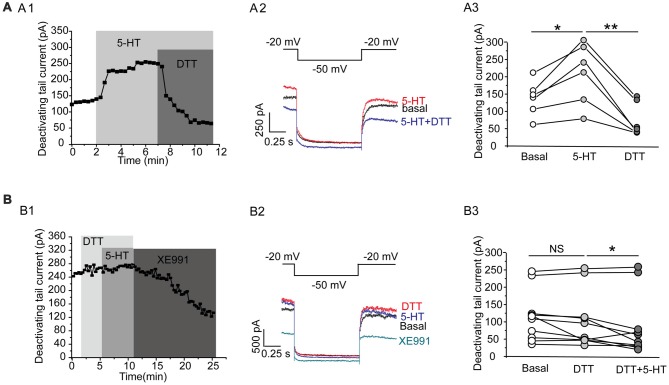
A redox mechanism is involved in 5-HT-mediated activation of Kv7.4 currents in DRN 5-HT neurons. **(A1)** 5-HT activation of Kv7.4 current was reversed by a reducing agent dithiothreitol (DTT). **(A2)** Voltage clamping protocol and the corresponding traces recorded from **(A1)**. **(A3)** Summarized data for experiments shown in **(A1)** (Wilcoxon Signed Ranks Test, *P < 0.05, **P < 0.01, *n* = 6, *N* = 4). **(B1)** Pretreatment with DTT (1 mM) had no effect on Kv7 currents but prevented the 5-HT-induced augmentation of the Kv7 currents. **(B2)** Voltage clamping protocol and representative current traces recorded from **(B1)**. **(B3)** Summarized data for experiments shown in **(B1)** (Wilcoxon Signed Ranks Test, *P < 0.05, *n* = 11, *N* = 5).

This effect of 5-HT is likely mediated by 5-HT_1A_ receptor because 5-HT_1A_ agonist 8-OH-DPAT induced a similar activation of the Kv7 currents (*N* = 8, **P = 0.004, paired samples test; Figure 5A), while 5-HT_1A_ receptor antagonist WAY-100635 blocked the effect of 5-HT in DRN 5-HT neurons of WT mice (*n* = 11, *P* = 0.328, Wilcoxon Signed Ranks Test; Figure 5B). It has been shown that substance P activates Kv7/M currents in DRG neurons through a Gi/Go pathway and a downstream redox mechanism (Linley et al., 2012). We next tested whether this mechanism is also involved in 5-HT-mediated activation of Kv7.4 currents in DRN 5-HT neurons. 5-HT activation of Kv7.4 currents was reversed by a reducing agent, dithiothreitol (DTT, 1 mM; *n* = 6, *P = 0.028, Wilcoxon Signed Ranks Test; Figure 6A), or prevented by DTT (*n* = 11, *P = 0.014, Wilcoxon Signed Ranks Test; Figure 6B). These results suggest that Kv7.4 is a target of 5-HT during the neuronal auto-inhibition, which possibly occurs through redox modification of the channel (Linley et al., 2012).

## Discussion

Here, we demonstrate that: (1) Kv7.4 is selectively expressed and exists as a major Kv7/M channel subunit in DRN 5-HT neurons and modulation of Kv7.4 (by fasudil) significantly affects (inhibits) the excitability of DRN 5-HT neurons; and (2) Kv7.4 is a target of 5-HT-mediated regulation in DRN 5-HT neurons, which is possibly through the cellular redox modification of the channel activity. It is well-established that serotonin plays an essential role in mood control, and regulating serotonergic neuronal activity produces a direct effect on several psychiatric disorders, such as anxiety and depression (Graeff et al., 1996; Nutt, 2002; Suri et al., 2015; Teissier et al., 2015).

5-HT neurons in the DRN are anatomically and functionally distinct. 5-HT neurons in DRN subfields project to different brain regions. For example, the rostral DRN projects to the caudate–putamen and substantia nigra, the middle DRN to the amygdala, whereas the caudal DR projects to the lateral and medial septum, ventral hippocampus, bed nucleus of the stria terminalis, locus coeruleus and hypothalamus. These subfield projections are likely involved in different behavior functions (Azmitia and Segal, 1978; Imai et al., 1986a,b; Datiche et al., 1995). In this study, we chose 5-HT neurons in the midline of vmDRN subdivisions of the DRN because in our immunohistochemistry study, this population of the neurons were most densely labeled by TPH, the marker of 5-HT neurons. However, in consideration of above mentioned diversity of DRN 5-HT neurons, it is not clear whether our present finding holds true for other subgroups of DRN 5-HT neurons. And since we did not present the behavior data in this study, we could not link our results to direct behavior significance, although ventromedial DRN is known to be involved in stress and anxiety. These issues are clearly interesting questions worthy of further study.

Our present finding that Kv7.4 is a powerful modulator of the serotonergic neuronal activity suggests a new strategy for treating psychiatric disorders linked to aberrant activity of 5-HT neurons. In fact, enhanced activity of K^+^ channels in VTA dopaminergic neurons has been shown to dampen the neuron activity and has an anti-depression effect (Wallace et al., 2009; Friedman et al., 2016). On the other hand and as noted in the introduction, blocking some K^+^ channels, such as SK channels (Crespi, 2010; Sargin et al., 2016), TREK1 channels (Ye et al., 2015) and Kir3/GIRK channels (Llamosas et al., 2015, 2017; Montalbano et al., 2015), substantially increases the activity of 5-HT-ergic neurons in the DRN and induces a significant antidepressant-like response. These findings promote an anticipated prospect of a close interaction between K^+^ channel function and the monoaminergic system in alleviating psychiatric disorders caused by aberrant neuronal activity.

Kv7.4 has restricted expression in the CNS; it is only expressed in discrete nuclei of the brainstem, including the midbrain (Kharkovets et al., 2000; Dalby-Brown et al., 2006), while other major Kv7 channel members, such as Kv7.2 and Kv7.3 subunits, are present in almost all brain regions examined thus far (Biervert et al., 1998). This selective expression presents Kv7.4 as a potential target for treatment of neuronal disorders involving aberrant activity of neurons. In a recent separate study, we found that fasudil was able to ameliorate the increased excitability of VTA DA neurons and the related depression-like behavior in a social defeat mouse model of depression (Li et al., 2017). Following the same line of thinking, we expect that the current finding that Kv7.4 is an important modulator of 5-HT neurons would be translated into novel Kv7.4-based treatments of diseases characterized by 5-HT neuronal overactivity, such as anxiety and fear (Korsgaard et al., 2005; Maier and Watkins, 2005). We have made some efforts to study Kv7.4 involvement in 5-HT neuron modulation in an *in vivo* setting, and the effect of fasudil on the firing of 5-HT neurons was observed using an *in vivo* extracellular recording method. However, our efforts were met with a few challenges: (1) as reported (Kocsis et al., 2006), the putative 5-HT neurons seemed very quiet and few were observed firing action potentials in an anesthetized mouse; (2) it is difficult to obtain a decent lasting recording without causing bleeding of DRN around the recording pipette which obscures the recordings. A method of direct DRN local pressure injection during single unit recording has been reported recently (Pernar et al., 2004). Further efforts will be made to correlate our findings with the *in vivo* electrophysiological study, and still furthermore, with the behavior study to be carried out.

We found that 5-HT activated Kv7.4 through a Gi/o-mediated and ROS-dependent pathway given that the channel activation was blocked by the reducing agent DTT. This modulatory pathway is similar to that shown in previous studies reporting that activation of PTX-sensitive Gi/o-coupled receptors (Somatostatin and substance P) can augment Kv7 currents in hippocampal neurons (Moore et al., 1988) and DRG neurons (Linley et al., 2012).

## Author Contributions

CZ and HZ: performed and planned experiments, analyzed and interpreted data and wrote the article; MS: performed part of electrophysiology experiments; YW: took care of transgenetic mice; XL: performed part of single-cell PCR; YZ: performed part of statistical analysis; XD: participated in planned experiments.

## Conflict of Interest Statement

The authors declare that the research was conducted in the absence of any commercial or financial relationships that could be construed as a potential conflict of interest.
